# Asperuloside Prevents Peri-Implantitis via Suppression of NF-κB and ERK1/2 on Rats

**DOI:** 10.3390/ph15081027

**Published:** 2022-08-20

**Authors:** Xinge Wang, Xutao Chen, Zhaoxin Zhang, Ji Chen, Zeyang Ge, Shitou Huang, Hongbo Wei, Dehua Li

**Affiliations:** State Key Laboratory of Military Stomatology & National Clinical Research Center for Oral Diseases & Shaanxi Engineering Research Center for Dental Materials and Advanced Manufacture, Department of Oral Implants, School of Stomatology, The Fourth Military Medical University, Xi’an 710032, China

**Keywords:** asperuloside, peri-implantitis, osteoclasts, NF-κB signaling, ERK1/2 signaling

## Abstract

Peri-implantitis is characterized by inflammatory cell infiltration and hyperactivation of the osteoclasts surrounding dental implants which can result in bone resorption and ultimately implant failure. Therefore, coordinating the activity of inflammatory response and bone-resorbing osteoclasts is crucial for the prevention of peri-implantitis. Asperuloside (ASP), an iridoid glycoside, has significant anti-inflammatory activities, suggesting the great potential in attenuating peri-implantitis bone resorption. A ligature-induced peri-implantitis model in the maxilla of rats was established, and the effects of ASP on preventing peri-implantitis were evaluated after four weeks of ligation using micro-CT and histological staining. RT-PCR, western blotting, tartrate-resistant acid phosphatase (TRAP), and immunofluorescent staining were conducted on osteoclasts to confirm the mechanisms of ASP on osteoclastogenesis. The results show that ASP could lead to attenuation of alveolar bone resorption in peri-implantitis by inhibiting osteoclast formation and decreasing pro-inflammatory cytokine levels in vivo. Furthermore, ASP could inhibit osteoclastogenesis by downregulating expression levels of transcription factors nuclear factor of activated T-cell (NFATc1) via restraining the activations of nuclear factor kappa beta (NF-κB) and the phosphorylation of extracellular signal-related kinase 1/2 (ERK1/2). In conclusion, ASP could significantly attenuate bone resorption in peri-implantitis via inhibition of osteoclastogenesis by suppressing NF-κB and ERK1/2 signaling pathways activations.

## 1. Introduction

Oral implants have become a popular treatment option for patients with partial or total edentulousness who want to improve their function and appearance [1,2]. Peri-implantitis is characterized by peri-implant mucositis and alveolar bone resorption [3], and is reported to take place in approximately 12–24% of dental implants [4,5]. Peri-implantitis has become the main reason for dental implants failure. Furthermore, it was reported that the morbidity of peri-implantitis increases with growing application of dental implants [6]. Experimental research on both humans and animals has shown that the plaque is a main etiological factor for peri-implantitis. Patients with a past of poor plaque control, severe periodontitis, and no routine maintenance after implant therapy have a higher risk for peri-implantitis development [3,7].

The pathogenic bacteria and their metabolites that adhere to dental implant surfaces may promote secretion of pro-inflammatory cytokines such as tumor necrosis factor-alpha and interleukins-1 and -6 (TNF-α, IL-1, and IL-6), which may induce a local inflammatory response that augments osteoclast formation and resorption activity, and eventually lead to peri-implant osteolysis and implant failure [8]. Prevention is a priority for peri-implantitis because there is still no universally recognized strategy to deal with peri-implantitis successfully and predictably [9]. However, current approaches to prevent peri-implantitis are mostly restricted to controlling dental plaques [10]. On the basis of the pathogenesis of peri-implantitis, reducing the secretion of pro-inflammatory cytokines and inhibiting excessive osteoclastogenesis might be one promising strategy for preventing peri-implantitis.

Osteoclasts, the principal cells with bone resorption function, are large multinucleated cells that develop from osteoclast precursor cells after a series of differentiation steps [11,12]. The NF-κB and mitogen activated protein kinases (MAPK) are crucial signaling pathways in osteoclastogenesis [13,14], once activated by receptor activator of nuclear factor kappa beta ligand (RANKL), they could induce the activations of c-Fos and NFATc1, and further promote osteoclast associated genes expression, such as ATPase H+-transporting V0 subunit d2 (Atp6v0d2), dendritic cell specific transmembrane protein (DC-STAMP), tartrate-resistant acid phosphatase (TRAP), cathepsin K (CTSK) and matrix metallopeptidase-9 (MMP-9), and eventually lead to osteoclast maturation [14,15]. Due to osteoclasts having a significant role in peri-implant bone resorption and implant loosening, drugs targeting inhibition of the osteoclastogenesis might be effective for peri-implantitis prevention. The main classes of anti-osteoclastogenesis drugs include biphosphonates, sclerostin antibodies, and strontium ranelate [16,17,18]. However, clinical research has shown that these medications do have certain negative effects [19,20,21]. Therefore, it is essential to identify new drugs for anti-osteoclastogenesis actions.

Natural compounds and their derivatives have received extensive attention recently due to the good efficacy and minimal toxic and adverse effects [22]. Asperuloside (ASP), an iridoid glycoside, is isolated from several plants belonging to the *Rubiaceae* or *Eucommiaceae* family, and has excellent anti-inflammatory and anti-oxidative effects. ASP has been extensively involved in research of some inflammatory diseases, including acute lung injury, uric acid nephropathy, and rheumatoid arthritis [23,24,25]. ASP was shown to cause a decrease in cartilage damage and accelerate the recovery of bone and joint activities in the treatment of rheumatoid arthritis [25]. Moreover, ASP could reduce the pro-inflammatory cytokines levels and lead to depression of the inflammation process via suppression of the activations of NF-κB and MAPK signaling pathways in macrophages in vivo and in vitro [23,26]. However, the impact of ASP on osteoclasts and osteolytic disorders is not well understood. Since NF-κB and MAPK signaling are vital in RANKL-mediated osteoclastogenesis [27,28]. It was hypothesized that ASP may possess preventive potential for peri-implantitis by downregulating pro-inflammatory cytokines levels and restraining osteoclastogenesis.

In this study, a ligature-induced peri-implantitis model in maxilla of rats was established to detect the effect of ASP on preventing peri-implantitis in vivo. Furthermore, in vitro experiments, osteoclasts were cultured to explore the effects and mechanisms of ASP on osteoclastogenesis and bone resorption function. Surprisingly, it was found that ASP could not only reduce the production of TNF-α, IL-1β, IL-6 and RANKL in peri-implant tissues and attenuate alveolar bone loss in the peri-implantitis model, but also could inhibit osteoclast differentiation and bone resorption activity by suppressing the activation of NF-κB and ERK1/2 signaling pathways in vitro, which suggest ASP could inhibit osteoclast formation by indirect suppression of osteoclastogenesis caused by downregulating pro-inflammatory cytokines levels, as well as the direct effects on osteoclast differentiation. As a result, ASP may be a prospective drug for peri-implantitis prevention.

## 2. Results

### 2.1. ASP Caused a Decrease in the Morbidity and Alveolar Bone Resorption of Peri-Implantitis

**Figure 1:**
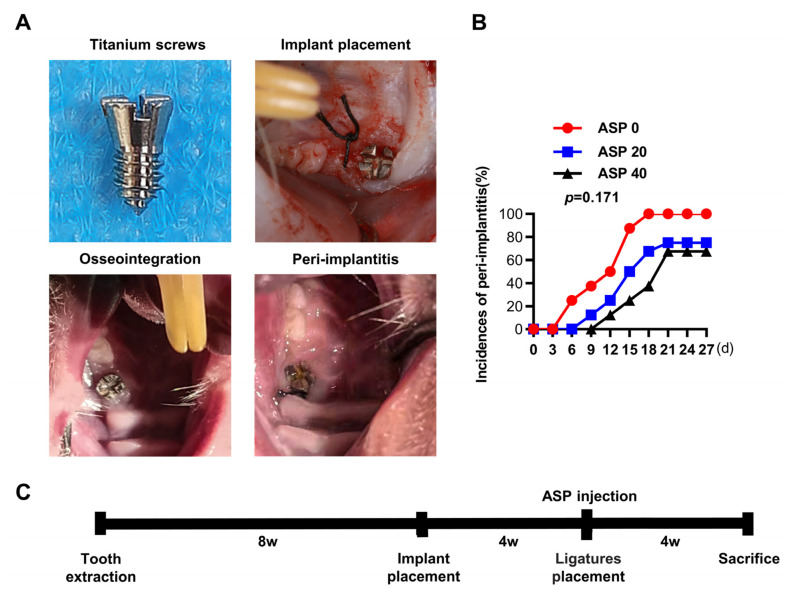
(**A**) Representative images and flow chart of animal experiments. (**B**) Morbidity of peri-implantitis in each group (*n* = 8). The correlation was determined using Pearson’s chi-squared test. (**C**) Schematic presentation of the timing of experimental design.

**Figure 2:**
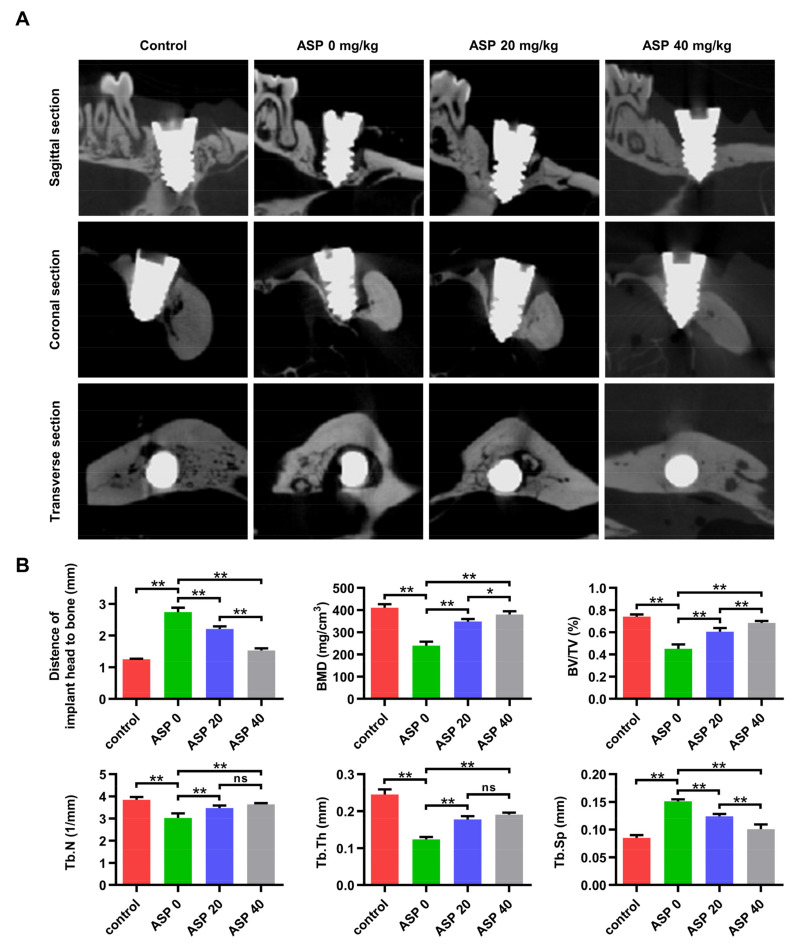
ASP led to a decrease in alveolar bone loss of peri-implantitis. (**A**) Maxilla Micro-CT images of each group. (**B**) Peri-implant bone loss level and bone morphometric parameters: Bone mineral density, bone volume/tissue volume, trabecular number, trabecular thickness, and trabecular separation (BMD, BV/TV, Tb.N, Tb.Th, and Tb.Sp, respectively) from every group (*n* = 5) were quantitated. ns: no significance, * *p* < 0.05, ** *p* < 0.01.

Four weeks after ligature induction of peri-implantitis, it was found that the earliest onset time of peri-implantitis for the ASP 0, 20, and 40 groups occurred on days 6, 9, and 12, respectively (Figure 1B). Furthermore, the morbidity of peri-implantitis dropped from 100% (8/8) in the ASP 0 group to 75% (6/8) in the ASP 20 group and 67.5% (5/8) in the ASP 40 group, but no statistically significance differences among different groups were noted (Figure 1B). The CT examination revealed that the titanium alloy screw implants in the control group had good bone integration, which direct contacted with the alveolar bone. Whereas, the ASP 0 group exhibited remarkable alveolar bone resorption around the implants. However, the alveolar bone absorption decreased significantly in the ASP 20 group and was nearly reversed in the ASP 40 group (Figure 2A). Compared with the ASP 0 group, ASP caused a distinct increase in BMD, BV/TV, Tb.N, and Tb.Th, while causing a decrease in the Tb.Sp, as revealed by bone morphometry analysis (Figure 2B). Taken together, ASP was found to delay the onset and cause a reduction in morbidity and alveolar bone loss of peri-implantitis dose-dependently. 

### 2.2. ASP Decreased the Inflammatory Tissues and the Osteoclast Quantity of Peri-Implantitis

**Figure 3:**
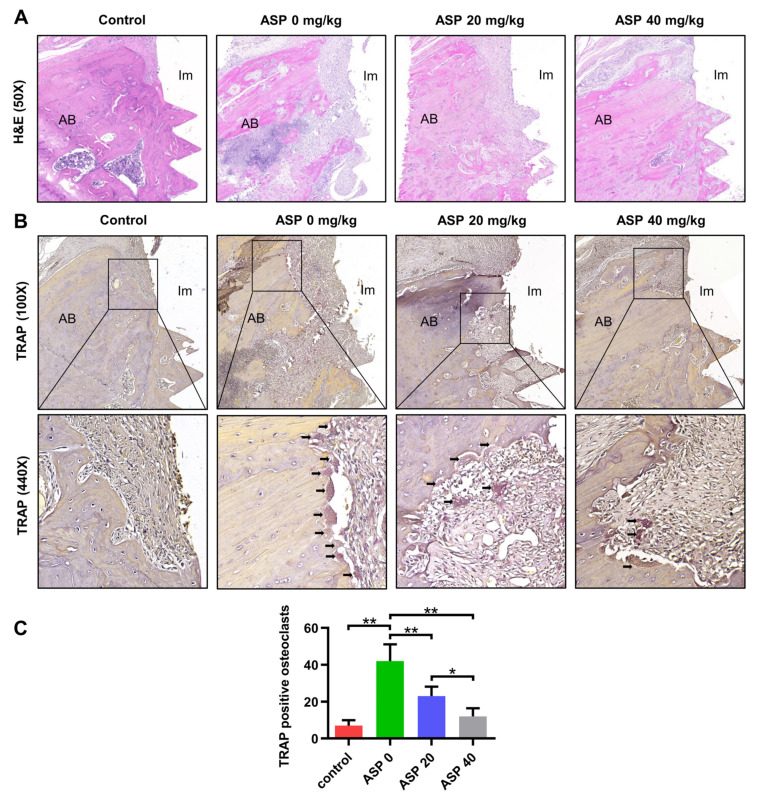
ASP caused a reduction in the inflammatory tissues and a decrease in the counts of osteoclasts in peri-implant tissues. (**A**) Illustrative H&E staining images of peri-implant tissues in every group. (**B**) Visualization of osteoclasts in peri-implant tissues from every group was achieved by TRAP staining (Black arrows indicate TRAP positive cells). (**C**) The TRAP positive osteoclasts were quantitated. *n* = 5, * *p* < 0.05, ** *p* < 0.01. AB, alveolar bone; Im, implant.

Hematoxylin and eosin (H&E) staining results revealed that almost no inflammatory tissues were found in the control group. Nevertheless, distinct inflammatory tissues around the implant were observed in the ASP 0 group, but the inflammatory tissues were obviously reduced in the ASP 20 group and were almost eliminated in the ASP 40 group (Figure 3A). In addition, TRAP staining revealed that ASP 20 and 40 groups have significantly fewer osteoclasts in their peri-implant tissues than the ASP 0 group (Figure 3B,C).

### 2.3. ASP Caused a Decrease of Pro-Inflammatory Cytokines and RANKL in Peri-Implant Tissues

**Figure 4:**
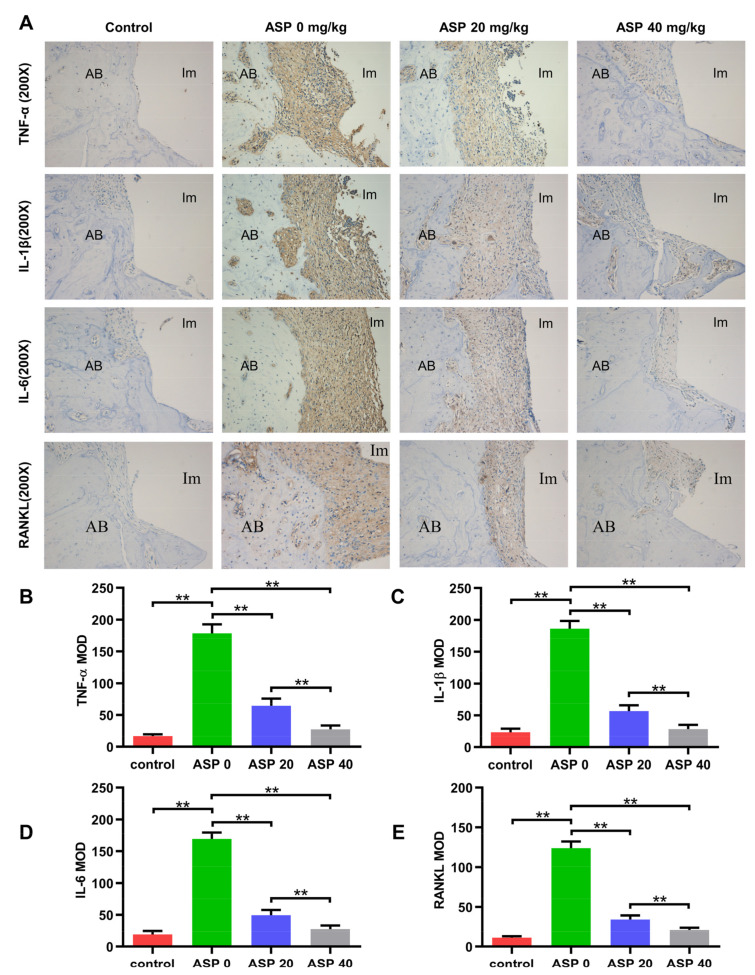
ASP treatment led to a decrease in the levels of TNF-α, IL-1β, IL-6 and RANKL in peri-implant tissues. (**A**–**E**) TNF-α, IL-1β, IL-6 and RANKL levels in peri-implant tissues from every group were evaluated by IHC staining. (MOD, mean optical density; AB, alveolar bone; Im, implant). *n* = 5, ** *p* < 0.01.

As shown in Figure 4A–E, immunohistochemical (IHC) staining showed that pro-inflammatory cytokines (TNF-α, IL-1β, IL-6) and RANKL levels increased significantly in the ASP 0 group relative to the control group. However, pro-inflammatory cytokines and RANKL levels decreased remarkably in the ASP 20 and 40 groups in contrast to the ASP 0 group. Furthermore, ASP treatment led to a dose-dependent and statistically significant decrease in the levels of TNF-α, IL-1β, IL-6 and RANKL.

### 2.4. ASP Inhibited RANKL-Induced Osteoclast Formation In Vitro

**Figure 5:**
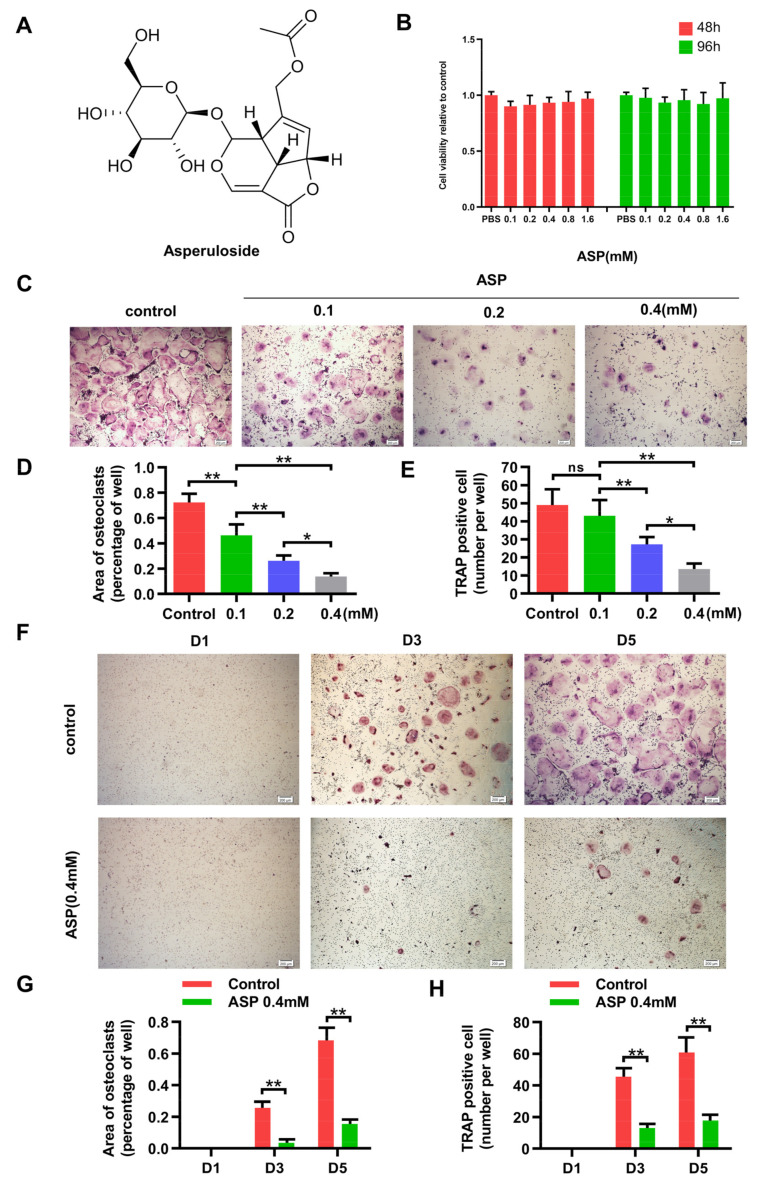
ASP downregulated RANKL-mediated osteoclast formation. (**A**) Chemical structure of ASP. (**B**) The cell viability of BMMs was assessed by CCK-8 kit. (**C**) TRAP staining imagines of osteoclasts treated with different concentrations of ASP. (**D**) Area and (**E**) number of TRAP positive osteoclasts were quantitated. (**F**) BMMs were treated with 0.4 mM ASP or PBS and TRAP staining was conducted on days 1, 3 and 5. (**G**) Area and (**H**) counts of osteoclasts were quantitated. Scale bar = 200 µm. *n* = 5, ns: no significance, * *p* < 0.05 and ** *p* < 0.01.

ASP reduced the RANKL levels and osteoclast counts in peri-implant tissues, but whether ASP directly inhibit osteoclast differentiation remains unclear. The in vitro impacts of ASP on osteoclast differentiation were assessed. The CCK-8 findings revealed that the cell viability of bone marrow macrophages (BMMs) was not influenced by ASP at the indicated concentration (Figure 5B). After five days induction by RANKL, multiple TRAP-positive mature osteoclasts were noted in the control group. Nevertheless, ASP treatment dose-dependently reduced the quantity and size of osteoclasts (Figure 5C–E). Moreover, after three days of RANKL induction, TRAP-positive osteoclasts began to fuse and form, whereas 0.4 mM ASP treatment caused a considerable suppression of osteoclast formation (Figure 5F–H). These discoveries indicated that ASP treatment could lead to a reduction in osteoclast size and fusion while inhibiting RANKL-induced osteoclast differentiation.

### 2.5. ASP Led to Inhibition of Bone Resorption Function of Osteoclasts In Vitro

**Figure 6:**
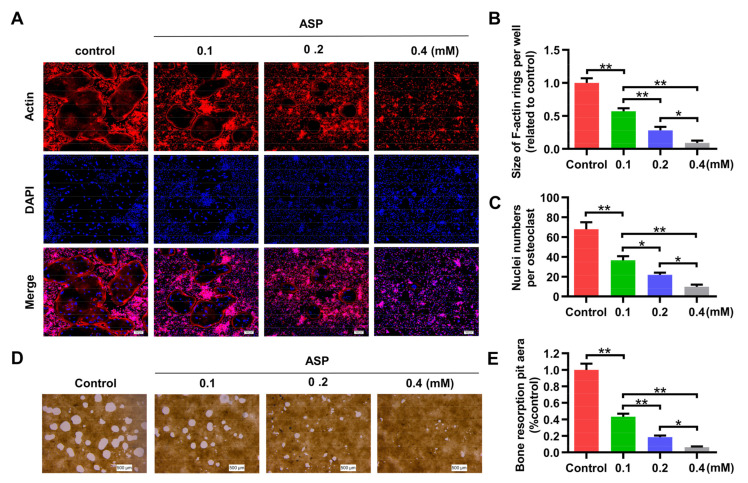
ASP inhibited bone resorption function of osteoclasts. (**A**) Representative fluorescence images of formed F-actin ring. Scale bar = 100 µm. (**B**) Mean F-actin ring areas of each group. (**C**) Number of nuclei per osteoclast from each group. (**D**) Representative micrographs of the resorption pits of osteoclasts from each group. Scale bar = 500 µm. (**E**) The percentage of resorption pit areas relative to control group. *n* = 3, * *p* < 0.05, ** *p* < 0.01.

F-actin is currently regarded as a critical structure for bone resorption function of osteoclasts. The impact of various concentrations of ASP on the F-actin ring were explored. As expected, ASP treatment markedly reduced the size of the F-actin ring (Figure 6A, B), which was in accordance with the impact of ASP on osteoclast size as shown in Figure 5D. Furthermore, osteoclasts contained fewer nuclei after the dose-dependent ASP treatment (Figure 6A,C). In addition, the capacity of osteoclasts treated with ASP to resorb the hydroxyapatite coating was dramatically suppressed dose-dependently (Figure 6D,E). These results revealed that ASP could cause effective inhibition of the resorption function of mature osteoclasts dose-dependently.

### 2.6. ASP Caused Downregulation of Osteoclast-Specific Gene Expression and Protein Expression

**Figure 7:**
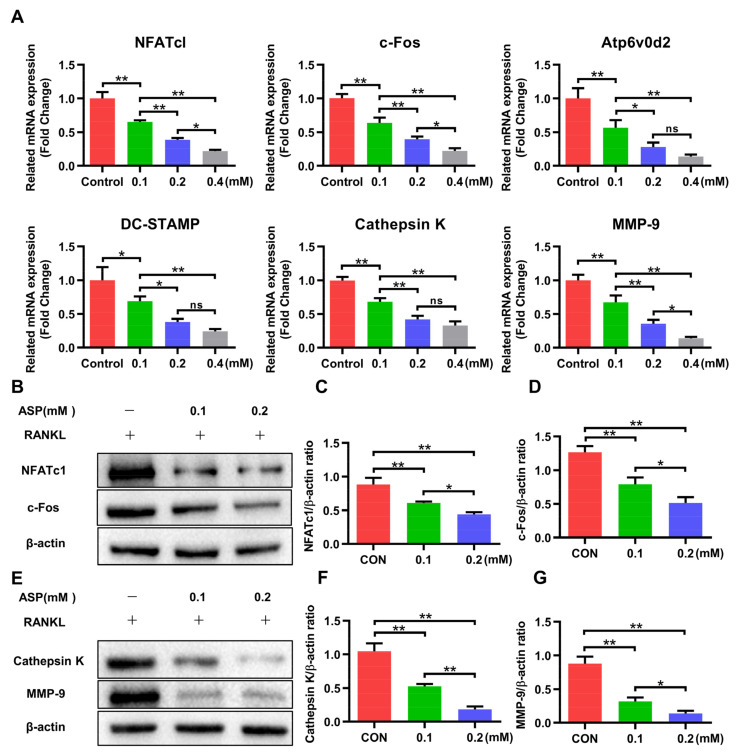
ASP downregulated expression levels of proteins and genes associated with osteoclasts (**A**) ASP downregulated the indicated osteoclast-specific gene expression. (**B**–**D**) Quantification of NFATc1 and c-Fos protein levels in various groups. (**E**–**G**) Quantification of CTSK and MMP-9 protein levels in various groups. *n* = 3, ns: no significance, * *p* < 0.05 and ** *p* < 0.01.

Since RANKL-induced overexpression of specific genes is associated with osteoclast differentiation, we used Real-Time PCR (RT-PCR) to figure out their expression changes after ASP treatment. Figure 7A showed that the gene expression of NFATc1, c-Fos, Atp6v0d2, DC-STAMP, CTSK, as well as MMP-9 were found to show a marked, dose-dependent decrease after 3 days of treatment with ASP. Consistent with the RT-PCR results, ASP led to a dose-dependent lowering of RANKL-stimulated protein expression levels of NFATc1and c-Fos (Figure 7B–D), as well as CTSK and MMP-9 (Figure 7E–G). To sum up, this study provided evidence that ASP might cause suppression of osteoclastogenesis by attenuating osteoclast-specific gene and protein expression in vitro.

### 2.7. ASP Attenuated the Activation of RANKL-Induced NF-κB and ERK1/2

**Figure 8:**
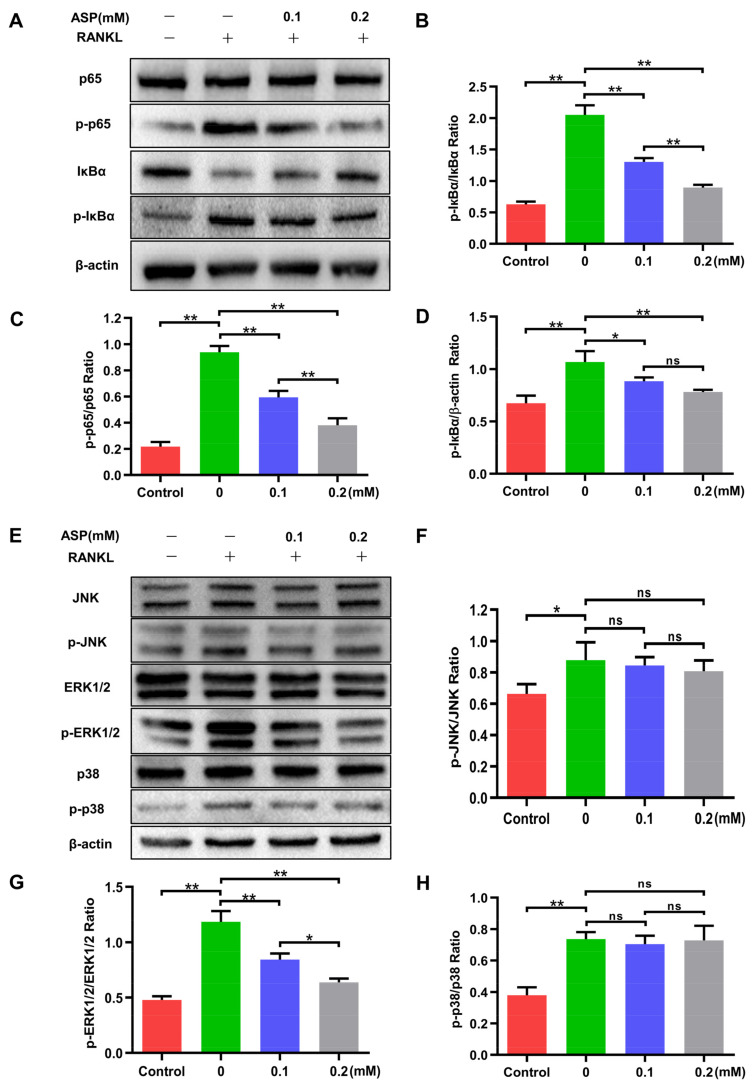
ASP attenuated the RANKL-induced NF-κB and ERK1/2 signaling pathways activations. (**A**–**D**) Effects of ASP on NF-κB pathway. (**E**–**H**) Effects of ASP on MAPK pathway. *n* = 3, ns: no significance, * *p* < 0.05 and ** *p* < 0.01.

NF-κB and MAPK pathways are considered extremely important in osteoclast formation. Thus, western blotting was conducted to assess the impacts of ASP on RANKL-induced activation of NF-κB and MAPK signaling pathways. It was found that ASP treatment led to a significant and dose-dependent decrease in both p65 and IκBα phosphorylation after RANKL stimulation for 30 min (Figure 8A–D). Moreover, ERK1/2 phosphorylation is markedly inhibited by ASP treatment relative to total protein, while phospho-JNK and phospho-p38 are not affected by ASP treatment (Figure 8E–H). Collectively, ASP suppressed RANKL-mediated NF-κB and ERK1/2 signaling pathways activations.

### 2.8. The ERK1/2 phosphorylation Agonist Caused Partial Reversal of the Inhibiting Effects of ASP on Osteoclastogenesis

**Figure 9:**
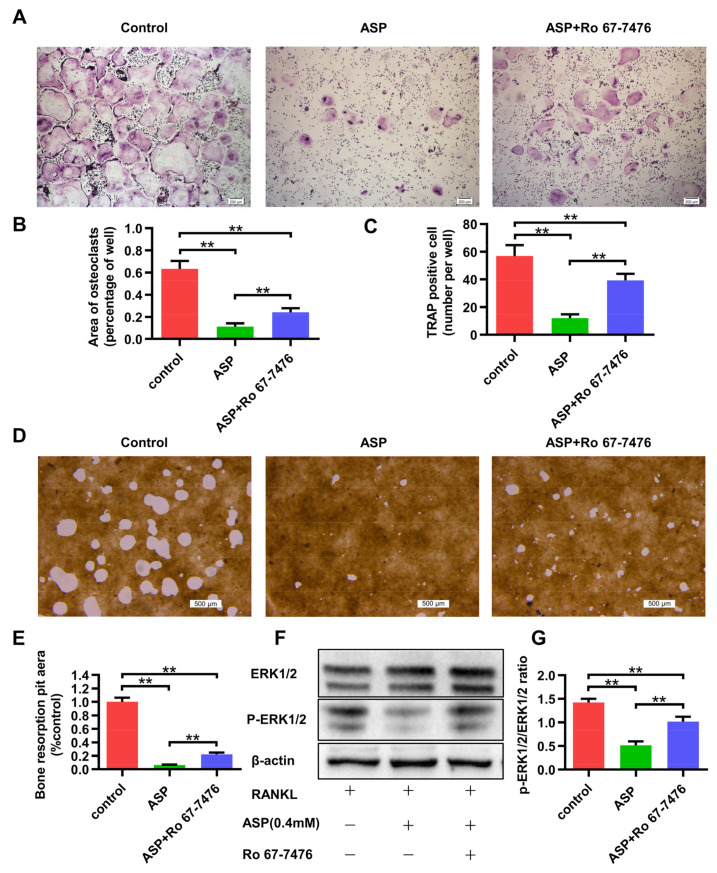
Ro 67-7476 treatment led to a partial reversal of the inhibitory effects of ASP on RANKL-mediated osteoclastogenesis. (**A**) BMMs were cultured for five days in osteoclastogenic medium with PBS, ASP (0.4 mM), and ASP (0.4 mM) with Ro 67-7476 (1 µM), and TRAP staining was conducted. Scale bar = 200 µm. (**B**) Area and (**C**) number of TRAP positive osteoclasts were quantitated. (**D**) BMMs were cultured for five days in 24-well bone resorption assay plates with osteoclastogenic medium with PBS, ASP (0.4 mM), as well as ASP (0.4 mM) with Ro 67-7476 (1 µM), and bone resorption pits were assessed by light microscope. Scale bar = 500 µm. (**E**) Bone resorption pit areas were quantitated. (**F**,**G**) BMMs were pre-treated with PBS, ASP, and ASP (0.4 mM) with Ro 67-7476 (1 µM) for 4 h and treated with RANKL for 30 min. Relative ERK1/2 and phosphor-ERK1/2 levels were quantitated using ImageJ software. *n* = 3, ** *p* < 0.01.

As previously mentioned, ASP suppressed osteoclastogenesis in vitro by inhibiting the activation of the ERK1/2 phosphorylation. To further verify these findings, whether Ro 67-7476 (ERK1/2 phosphorylation agonist) with ASP treatment can rescue the suppression on osteoclast differentiation was examined. The TRAP staining results demonstrated that ASP (0.4 mM) treatment alone resulted in considerable suppression of osteoclastogenesis. However, osteoclasts were shown to be more numerous and larger in the group treated with ASP (0.4 mM) and Ro 67-7476 (1 µM) (Figure 9A–C). Subsequently, we confirmed that the suppression of bone-resorptive function of osteoclasts by ASP (0.4 mM) could be reversed by Ro 67-7476 (1 µM) to some extent (Figure 9D,E). Moreover, western blotting results revealed that Ro 67-7476 (1 µM) could rescue inhibition of ERK1/2 phosphorylation partially induced by ASP (0.4 mM) (Figure 9F,G). As a result, the ERK1/2 phosphorylation agonist was found to partially rescue the suppression of osteoclast formation and bone resorption activity caused by ASP treatment.

## 3. Discussion

Peri-implantitis is one of the inflammatory bone lysis diseases, which has become a major threat to implant health. Accordingly, inhibiting excessive inflammatory response and bone-resorption activity of osteoclasts might be one promising strategies for preventing peri-implantitis. ASP is a type of iridoid glycoside extracted from *Rubiaceae*, *Eucommiaceae* and other plants. Recent pharmacological research has revealed that ASP has anti-inflammatory, anti-oxidant, immunomodulatory, anti-obesity, anti-cancer, anti-hypertension and many other pharmacological properties [29,30,31,32,33,34]. Asperuloside has attracted extensive attention from scholars because it has been shown to possess excellent effects on suppressing inflammation in many studies [23,24,26], but little is known about its pharmacological effects on peri-implantitis. In this study, we found that ASP reduced the incidence of peri-implantitis in vivo, and suppressed alveolar bone resorption around the implant via causing a decrease in the numbers of osteoclasts. Furthermore, our studies revealed that ASP could inhibit osteoclast differentiation as well as bone resorption activity by inhibiting NF-κB and ERK1/2 signaling pathways activations.

To verify the impact of ASP in preventing peri-implantitis in vivo, a peri-implantitis model was established via placement of 5-0 silk ligatures around the implants for the accumulation of dental plaques and pathogenic bacteria. This method is widely used to construct peri-implantitis models because it can cause rapid development of inflammation and a strong immunological response, and further cause excessive osteoclast formation and bone resorption that eventually results in peri-implantitis [35,36,37]. In this research, it was found that ASP led to a delay in the onset and a decrease in the incidence of peri-implantitis on rats. Micro-CT studies demonstrated that ASP protected against peri-implantitis bone resorption dose-dependently. The effects of ASP on decreasing bone loss and improvement in bone-implant contact were further verified by histological data of H&E staining. Furthermore, TRAP staining results revealed that ASP could lead to inhibition of in vivo osteoclastogenesis. Thus, our findings showed that ASP could produce a reduction in peri-implant bone loss in vivo by causing a reduction in osteoclast formation.

Moreover, our study revealed that ASP treatment reduced TNF-α, IL-1β, IL-6 and RANKL levels in peri-implant tissues, which were consistent with previous studies [23,26]. TNF-α, IL-1, and IL-6 have been identified as the most essential pro-inflammatory cytokine biomarkers and are strongly linked to the onset, development, and clinical consequences of peri-implantitis [38,39]. Significant raising of TNF-α, IL- 1β, and IL-6 in inflammatory tissues could not only facilitate the promotion of osteoclast formation and activation, but also lead to induction of RANKL secretion by other cells including osteoblasts to motivate osteoclastogenesis [40,41,42,43]. The discovery that ASP could lead to inhibition of the generation of TNF-α, IL-1β, as well as IL-6 in peri-implant tissues partially explained ASP-induced indirect suppression of osteoclastogenesis in inflammatory conditions.

To further assess the effects of ASP on osteoclast differentiation as well as bone resorption activities, the influence of ASP on osteoclastogenesis in vitro experiments was examined. ASP treatment led to a restraint of RANKL-mediated osteoclast differentiation, and decline in osteoclast size without causing any evident cytotoxicity. Moreover, ASP caused inhibition of the development of the F-actin ring and a decrease in osteoclasts’ capability to resorb bone. NFATc1 and c-Fos are generally considered as vital transcription factors for osteoclastogenesis [44,45,46]. It was found that ASP causes a significant reduction in mRNA and protein levels of NFATc1 and c-Fos by the stimulation of RANKL dose-dependently. In addition, ASP also led to a reduction in levels of NFATc1 responsive genes, such as Atp6v0d2, DC-STAMP, MMP-9, and CTSK. DC-STAMP and Atp6v0d2 were discovered to be essential for osteoclast fusion [47,48]. CTSK and MMP-9 are characteristic bone absorption proteins expressed in osteoclasts specifically and play a critical role in bone resorption function. Similarly, ASP led to a decline in RANKL-stimulated CTSK and MMP-9 mRNA and protein levels. Collectively, these findings suggested that the anti-osteoclastogenic effects of ASP are mediated in part by the direct suppression of NFATc1 signaling.

NF-κB and MAPK signaling pathways are critical to activate NFATc1, which leads to RANKL-induced osteoclast formation, as many studies have shown [27,28]. The recruitment of tumor necrosis factor receptor-associated factor (TRAF6) is induced by RANKL which then promotes the activation of NF-κB, ERK1/2, p38 mitogen-activated protein kinase (p38), and c-jun N-terminal kinase (JNK). Then several transcription factors are activated and subsequently activate the NFATc1. And NFATc1 stimulates the expression of osteoclast-specific genes and stimulates the differentiation as well as fusion of osteoclast precursor cells into mature, multinucleated osteoclasts [44,45]. It has been verified that ASP can cause a depression of the inflammatory process by inhibiting NF-κB and MAPK signaling pathways activations in macrophages [23,26]. Consistently, our findings showed that ASP treatment led to suppression of RANKL-induced NF-κB activation by dose-dependently suppressing IκBα phosphorylation and degradation. Previous research has shown that phosphorylation of ERK1/2 is critical for cell proliferation, survival, and differentiation of osteoclast precursor cells [49,50]. Furthermore, phospho-ERK1/2 activation enhances the expression of NFATc1 [51]. Our results showed that the ERK1/2 phosphorylation dramatically decreases after treatment with ASP, but no alterations in the phosphorylation of p38 and JNK occurred. An ERK1/2 phosphorylation agonist (Ro 67-7476) was used to co-culture BMMs with ASP to further demonstrate the suppressive function of ASP on ERK1/2 phosphorylation [52]. The agonist led to partial rescue of the suppressive influence on ERK1/2 phosphorylation by ASP, and promotion of osteoclast formation as well as bone resorption activity as expected. Consistently, Ro 67-7476 could lead to an increase in the protein expression of phospho-ERK1/2 that had been suppressed by ASP. Thus, it may be stated that ASP suppresses osteoclastogenesis via restraint of ERK1/2 phosphorylation. To summarize, our findings suggested that ASP may restrain NFATc1 expression by downregulating NF-κB and ERK1/2 signaling pathways activations.

**Figure 10:**
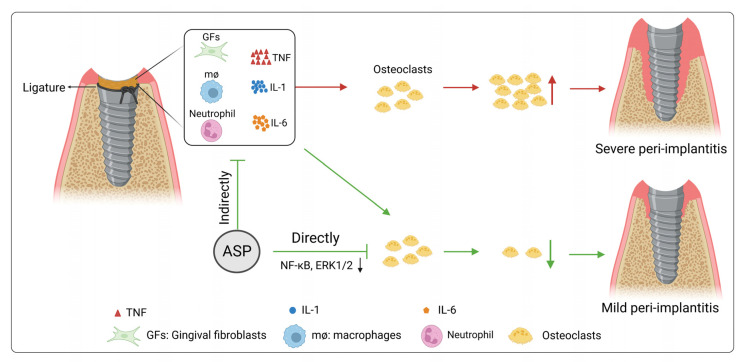
A proposed model illustrating the effects of ASP on peri-implantitis prevention.

In fact, this study revealed for the first time that ASP could suppress osteoclast differentiation, formation of F-actin ring, and bone resorption function of osteoclasts by direct inhibition of NF-κB and ERK1/2 signaling pathway. Moreover, ASP could indirectly inhibit osteoclast formation via reducing TNF-α, IL-1β, IL-6 and RANKL levels in peri-implant tissues. Corresponding to the direct and indirect function of ASP in osteoclastogenesis, ASP could lead to prevention of peri-implantitis by causing a decrease in alveolar bone resorption (Figure 10).

Our findings indicate that ASP may be a prospective drug choice for peri-implantitis prevention. However, there are still some limitations in this study. Firstly, drug delivery assessment should be studied before its potential clinical application to the prevention for peri-implantitis in the future. Secondly, the systemic administration of ASP has some disadvantages, such as high dosage, and some systemic effects. In fact, the topical or local administration is easier for the dentists and patients. Hence, we will use sustained release material loaded ASP for local treatment of peri-implantitis in the following experiments, so that it has the chance to be promoted in clinical application.

## 4. Materials and Methods

### 4.1. Materials and Reagents

Machined, smooth surface titanium alloy (Ti-6Al-4V) screws (3.5 × 1.6 mm) were obtained from Northwest Institute for Nonferrous Metal Research (Xi’an, China), and were cleaned and degreased in a trichloroethylene bath without specific surface pretreatments. CCK-8 assay kit was procured from Beyotime Institute of Biotechnology (Shanghai, China). Primary antibodies against p65, phospho-p65, IκBα, phospho-IκBα, NFATc1, c-Fos, and β-actin were acquired from Abcam (Cambridge, UK). Primary antibodies against JNK, phospho-JNK, ERK1/2, phospho-ERK1/2, p38, phospho-p38, MMP-9, and cathepsin K were obtained from Cell Signaling Technology (Danvers, MA, USA). IL-6, TNF-α, and IL-1β, antibodies were procured from Santa Cruz Biotechnology (Dallas, TX, USA). RANKL antibodies were acquired from Proteintech (Chicago, IL, USA). Macrophage colony-stimulating factor (M-CSF) and RANKL were bought from PeproTech (Rocky Hill, NJ, USA). The RNeasy Mini Kit was acquired from Takara (Otsu, Japan). The TRAP staining kit was obtained from Sigma-Aldrich (St. Louis, MO, USA). α-MEM and Fetal bovine serum (FBS) were acquired from Gibco (Rockford, IL, USA). ASP (purity > 98%; Figure 5A) was obtained from Shanghai Shifeng Biotechnology (Shanghai, China), diluted in phosphate buffer saline (PBS) and thereafter in α-MEM medium. Ro 67-7476 was purchased from MedChemExpress (Shanghai, China).

### 4.2. Animal Experiments

**Figure A1:**
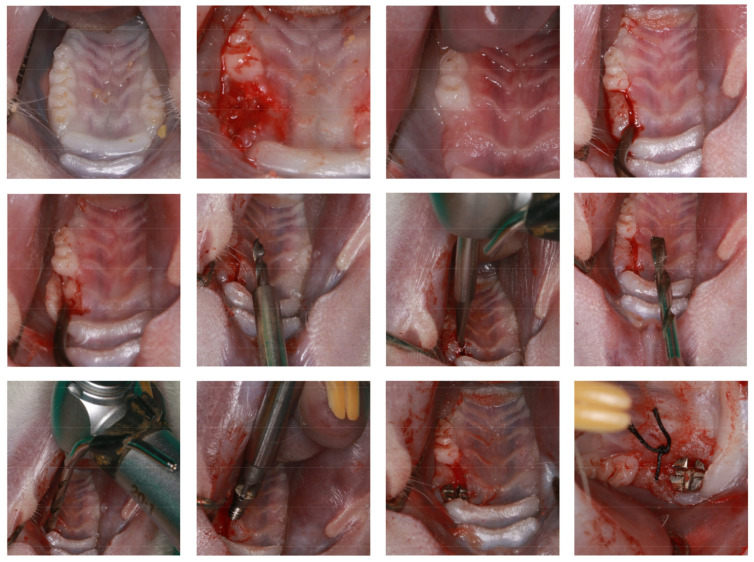
Photographic images of implantation surgery.

The Institutional Animal Ethics Committee of The Fourth Military Medical University permitted this study (Approval Code: 20210355) and all treatments to the animals were performed in accord with ARRIVE guidelines. First, the preventive potential of ASP for peri-implantitis was assessed in vivo. Forty 2-month-old male Sprague-Dawley rats (180–200 g) were fed at 20~24 °C. Pentobarbital sodium was injected intraperitoneally for general anesthesia and 0.5% lidocaine was used for local anesthesia. The left upper first molars were extracted for the first step, and the implantation surgery was performed after two months of wound healing. Titanium alloy screws (3.5 × 1.6 mm) were implanted in the first molar of left upper. Four weeks were required to achieve osseointegration after implant insertion. Four weeks later, nine rats were removed from the experiment (three rats died after surgery and six rats underwent implant loosening). Therefore, 31 rats (550–600 g) were allocated into four groups randomly: 1. control group (*n* = 7), 2. ASP 0 group (*n* = 8), 3. low dose ASP 20 group (*n* = 8, 20 mg/kg ASP), and 4. high dose ASP 40 group (*n* = 8, 40 mg/kg ASP). Subsequently, peri-implantitis were induced in the ASP 0, 20, and 40 groups via the placement of 5-0 silk ligatures around the head of the implants after which ligatures were kept for four weeks without intervention. At the same time, dosages of 20 and 40 mg/kg ASP dissolved in physiological saline were injected intraperitoneally and classified as the ASP 20 and 40 groups respectively, and the same dose of physiological saline was injected intraperitoneally for the ASP 0 and control groups. Fourteen injections were given in total every second day. Every three days, the implant mobility as well as probing depth of the implant were checked to identify peri-implantitis when implant looseness or probing depth > 0.5 mm was found. Four weeks later, ligatures were removed and all the animals were euthanized. The maxillae were dissected and were stabilized in 4% paraformaldehyde. The experimental procedure is shown in Figure 1A,C. Photographic images of implantation surgery were shown in Figure A1.

### 4.3. Micro-CT Scanning

Maxillae were scanned by a micro-CT (QuantumGX2, PerkinElmer, Waltham, MA, USA) at a 90 kV voltage and 80 µA current. Three-dimensional (3D) images were analyzed with Siemens Research Workplace software (Siemens). To analyze peri-implant bone loss, the distances between the most coronal position of marginal bone and the apical point of the head of the implant were measured on the distal, mesial, buccal, and palatal locations. BMD, Tb.Th, BV/TV, Tb.Sp and Tb.N were quantified to assess bone mass.

### 4.4. H&E Staining and TRAP Staining

After micro-CT examination, the maxilla samples (*n* = 5) were decalcified in 10% ethylenediaminetetraacetic acid solution for 30 days. Implants were manually removed from the maxillae. The samples were paraffin-embedded, sectioned, and H&E stained. Subsequently, TRAP staining was conducted for osteoclast identification, TRAP-positive multinucleated cells (≥3 nuclei) were recognized as osteoclasts.

### 4.5. Immunohistochemistry Staining

Immunohistochemistry staining was performed to detect pro-inflammatory cytokine (TNF-α, IL-1β, IL-6) as well as RANKL expression levels in peri-implant tissues. RANKL, TNF-α, IL-1β and IL-6 were immunolocalized after incubation with specific primary antibodies. Randomly, 5 targeted areas around the implant were selected for quantification of the integrated optical density (IOD) of positive staining by Image-pro plus 6.0 software, and to count the mean optical density (MOD = accumulated IOD of targeted areas/sum of targeted areas).

### 4.6. Bone Marrow-Derived Macrophages (BMMs) Culture and Cell Viability Assay

Bone marrow cells were acquired from tibias and femurs of 2-week-old male Sprague-Dawley rats and were cultured in complete α-MEM medium (supplemented with 1% penicillin/streptomycin and 10% FBS) containing 30 ng/mL M-CSF overnight. Then, non-adherent cells were obtained and cultured in complete α-MEM medium for 48 h. Adherent cells were obtained as BMMs. Cell viability was evaluated by the CCK-8 assay. BMMs (10^4^ cells/well) were inoculated in 96-well plate for 24 h prior to co-incubation with varying doses of ASP (0–1.6 mM) for 48 and 96 h with 30 ng/mL M-CSF. Optical density (OD) was determined at 450 nm.

### 4.7. Cell Culture and TRAP Staining Assay

BMMs (1.0 × 10^6^ cells/well) were treated without or with ASP (0.1, 0.2, 0.4 mM) in 6-well plate for five days and with M-CSF (30 ng/mL) as well as RANKL (100 ng/mL). Cells were fixed and stained with TRAP solution as per the manufacturer’s guidelines. TRAP-positive multinucleated cells (≥3 nuclei) were identified as osteoclasts, and their counts as well as distributed areas were quantitated by microscopy.

### 4.8. Fibrous Actin (F-Actin) Ring Staining Assay

BMMs (1.0 × 10^6^ cells/well) were inoculated in 6-well plate and cultured without or with ASP (0.1, 0.2, 0.4 mM) supplemented with M-CSF (30 ng/mL) as well as RANKL (100 ng/mL) for five days. Fixed cells were incubated for 1 h with rhodamine-conjugated phalloidin diluted (1:100) at 37 °C, washed in PBS, labeled with 4′,6-diamidino-2-phenylindole (DAPI), and images were recorded using an Olympus FV1000 Laser Scanning Confocal Microscope.

### 4.9. In Vitro Hydroxyapatite Resorption Assay

BMMs (1.0 × 10^5^ cells/well) were inoculated in 24-well Hydroxyapatite Resorption Assay plates (Cosmo Bio, JAP) and treated without or with ASP (0.1, 0.2, 0.4 mM) supplemented with M-CSF (30 ng/mL) as well as RANKL (100 ng/mL) for five days. Subsequently, cells were sterilized with 5% (*w*/*v*) sodium hypochlorite after which the plates were washed using water and dried. Light microscope was performed to photograph the resorption pit area per well, which was then calculated using Image J software.

### 4.10. Real-Time PCR

BMMs (1.0 × 10^6^ cells/well) were inoculated in 6-well plate, and cultured without or with ASP (0.1, 0.2, 0.4 mM) followed by the stimulation with M-CSF (30 ng/mL) as well as RANKL (100 ng/mL) for three days. Extraction of total RNA was conducted using the RNA Extraction Kit and cDNA synthesized by reverse transcription from 20 µL of total RNA using the PrimeScriptTMRT Master Mix (Takara Bio, Inc., Otsu, Japan). Target genes expression were assayed by RT-PCR using an ABI 7500 Fast Real-Time PCR System (Foster City, CA, USA). Primers used are shown in Table 1; β-actin was used as the internal reference.

### 4.11. Western Blotting Assay

BMMs (1.0 × 10^6^ cells/well) were seeded in 6-well plate with complete α-MEM supplemented with M-CSF (30 ng/mL) and RANKL (100 ng/mL) for 3 days, either without or with ASP (0.1, 0.2 mM), to examine the impact of ASP on protein expression of NFATc1, c-Fos, cathepsin K and MMP-9. To explore the exact signaling pathway that ASP affected, BMMs (1.0 × 10^6^ cells/well) were seeded in 6-well plates with complete α-MEM as well as M-CSF (30 ng/mL) for 24 h and subsequently pretreated without or with ASP (0.1, 0.2 mM) for 4 h, and finally treated with RANKL (100 ng/mL) for 30 min. Protein levels of NF-κB and MAPK signaling pathway were assessed. Total proteins for each sample were extracted and quantified. Equal amounts of protein (20 µg) were placed into each well of a polyacrylamide-sodium dodecyl sulfate (10% SDS) gel before being transferred to a PVDF membrane. Overnight incubation of membranes with suitable primary antibody (1:1000 dilution) was conducted at 4 °C after being blocked by 5% skim milk. The PVDF membrane was then incubated in the presence of HRP-conjugated secondary antibodies (1:5000 dilution) for one hour at room temperature following three TBST washings. Finally, the chemiluminescence signals were detected using the ECL reagent. Quantitative analyses of proteins were conducted using ImageJ software.

### 4.12. Statistical Analysis

Data are shown as mean ± standard deviation (SD). In vitro experiments were independently repeated at least thrice. To ensure that parametric tests can be performed, the Shapiro-Wilk test for normality was employed to examine all of the data firstly. The correlation was assessed by Pear-son’s chi-squared test. An unpaired t-test was used for the comparisons between two groups. Comparisons among multiple groups were conducted by one-way ANOVA and Tukey’s post hoc tests. Analyses were conducted by the SPSS software for Windows (version 26.0, SPSS Inc., Chicago, IL, USA). Results with *p* < 0.05 were considered statistically significant.

## 5. Conclusions

In conclusion, this research shows that ASP has anti-osteoclastogenesis and anti-bone-resorption effects. Moreover, ASP exhibits great potential to decrease inflammatory bone loss and prevent peri-implantitis in rats. Thus, our findings may not only provide a novel strategy for preventing peri-implantitis, but also broaden the application scope of ASP.

## Figures and Tables

**Table 1:** Primer sequences for Real-time PCR.

Gene Name	Forward Sequences 5′–3′	Reverse Sequences 5′–3′
NFATc1	TCCGTCCATACTGCCAGAGGTG	GGATTGTGCTGGAGAGGTCGTTAC
c-FOS	CCTTCACCCTGCCTCTTCTCAATG	AGCCTTCAGCTCCATGTTGCTAATG
DC-STAMP	GCCTCTTCCTGAAGCGATTCCTG	GGCACCTCTCCTCTTCATCAAACAG
Atp6v0d2	AGCCAGCCTCCTAACTCAGCAG	GAGCCAGGAAGTTGCCATAGTCAG
Cathepsin K	TGGCTGTGGAGGCGGCTATATG	CGGGTAAGCGTCTTCAGAGTCAATG
MMP-9	CCCATGTCACTTTCCCTTCACCTTC	CGATAACCATCCGAGCGACCTTTAG
β-actin	AGGAGTACGATGAGTCCGGC	CGCAGCTCAGTAACAGTCCG

**Figure A2:**
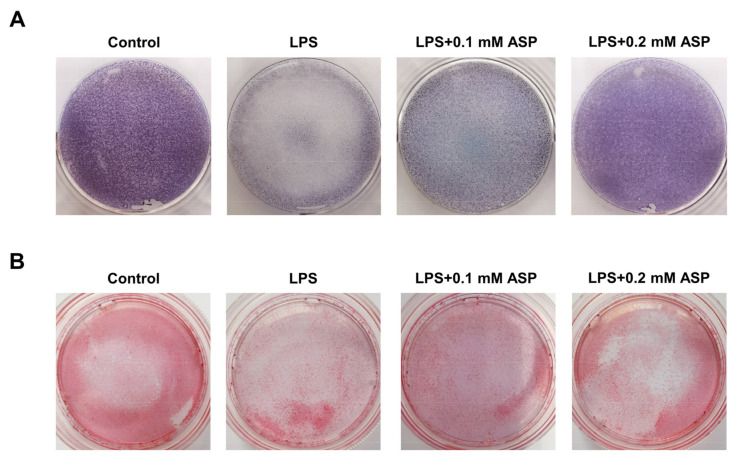
ASP suppressed the harmful effect of LPS on osteogenesis function of MC3T3-E1 cells. (**A**) ALP staining was conducted to detect the expression level of ALP in each group. (**B**) Alizarin red staining was performed to detect the mineralization function of MC3T3-E1 cells in each group.

## Data Availability

Data is contained within the article.
